# A Bayesian Reanalysis of the Overall and Sex-Disaggregated Results of the Neonatal Oxygenation Prospective Meta-Analysis (NeOProM)

**DOI:** 10.3390/antiox13050509

**Published:** 2024-04-24

**Authors:** Maurice Jacob Huizing, Tamara Maria Hundscheid, František Bartoš, Eduardo Villamor

**Affiliations:** 1 Division of Neonatology, MosaKids Children’s Hospital, Maastricht University Medical Center (MUMC+), Research Institute for Oncology and Reproduction (GROW), Maastricht University, 6202 AZ Maastricht, The Netherlands; 2 Department of Psychology, University of Amsterdam, 1001 NK Amsterdam, The Netherlands

**Keywords:** extremely preterm infants, neonatal oxygenation prospective meta-analysis, pulse oximeter saturation, mortality, necrotizing enterocolitis, retinopathy of prematurity, bronchopulmonary dysplasia

## Abstract

Data from the Neonatal Oxygenation Prospective Meta-analysis (NeOProM) indicate that targeting a higher (91–95%) versus lower (85–89%) pulse oximeter saturation (SpO_2_) range may reduce mortality and necrotizing enterocolitis (NEC) and increase retinopathy of prematurity (ROP). Aiming to re-evaluate the strength of this evidence, we conducted a Bayesian reanalysis of the NeOProM data. We used Bayes factors (BFs) to evaluate the likelihood of the data under the combination of models assuming the presence vs. absence of effect, heterogeneity, and moderation by sex. The Bayesian reanalysis showed moderate evidence in favor of no differences between SpO_2_ targets (BF_10_ = 0.30) in death or major disability, but moderate evidence (BF_10_ = 3.60) in favor of a lower mortality in the higher SpO_2_ group. Evidence in favor of differences was observed for bronchopulmonary dysplasia (BPD) (BF_10_ = 14.44, lower rate with lower SpO_2_), severe NEC (BF_10_ = 9.94), and treated ROP (BF_10_ = 3.36). The only outcome with moderate evidence in favor of sex differences was BPD. This reanalysis of the NeOProM trials confirmed that exposure to a lower versus higher SpO_2_ range is associated with a higher mortality and risk of NEC, but a lower risk of ROP and BPD. The Bayesian approach can help in assessing the strength of evidence supporting clinical decisions.

## 1. Introduction

The Oxygen Paradox states that while oxygen is essential for aerobic life forms, it is also inherently dangerous to those same life forms [1]. Arguably, the moment of life in which this paradox manifests itself in a more pronounced way is the transition from intrauterine to the extrauterine life. At birth, a newborn is exposed to the oxidative shock of transitioning from the relative hypoxia of fetal life to the atmospheric normoxia of postnatal life [2,3,4]. While this transition is generally smooth in term infants, because they have adequate antioxidant defenses, this is not the case in premature infants. Furthermore, the physiologically hypoxic intrauterine environment is a major stimulus for the development of organs and systems [5,6]. Preterm birth disrupts this physiological development, forcing immature organs and systems to assume their physiological functions too early. This alters the type of signals and stimuli that these organs and systems will receive for their subsequent development. Two other aspects need to be taken into account. The first is that an environment of oxidative stress may already have been induced by the pathological condition, or endotype, responsible for preterm birth [7]. The second is that therapeutic interventions, such as oxygen supplementation, mechanical ventilation, or parenteral nutrition, together with postnatal exposure to infectious inflammatory processes, may substantially increase the oxidative stress load [2,3,4]. Saugstad coined the term “oxygen radical disease of neonatology” in 1981, proposing that oxidative stress has a spectrum of effects in the neonate and might play a role in the pathogenesis of many complications of prematurity, including bronchopulmonary dysplasia (BPD), retinopathy of prematurity (ROP), or necrotizing enterocolitis (NEC) [8].

Oxygen therapy remains a difficult conundrum in neonatal care, as efforts to reduce the complications associated with hyperoxemia and oxidative stress in extremely preterm infants (i.e., gestational age below 28 weeks) may affect their survival [9,10]. The best available evidence on what range of pulse oximeter saturation (SpO_2_) should be targeted for extremely preterm infants from birth or shortly thereafter comes from the Neonatal Oxygenation Prospective Meta-analysis (NeOProM) Collaboration [11]. This prospective meta-analysis used harmonized individual participant data from five randomized controlled trials (RCTs) involving approximately 5000 infants [12,13,14,15,16,17]. The RCTs were the SUPPORT (Surfactant, Positive Pressure, and Oxygenation Randomized Trial), the three (Australia, New Zealand, and United Kingdom) BOOST II (Benefits of Oxygen Saturation Targeting II), and the COT (Canadian Oxygen Trial) [12,13,14,15,16,17]. The five trials included similar populations of extremely preterm infants, compared the same target SpO_2_ ranges, used similarly modified investigational oximeters to mask for allocation of the groups, and investigated the same short- and long-term outcomes. The NeOProM data showed that targeting a lower SpO_2_ range (85–89%) versus a higher SpO_2_ range (91–95%) increased mortality with a risk ratio (RR) of 1.17 and a 95% confidence interval (CI) of 1.04 to 1.31 (*p*  =  0.01). The lower saturation range was also associated with an increased risk of developing severe NEC (RR 1.33, 95% CI 1.10 to 1.61, *p* = 0.003), but decreased the risk of developing severe ROP (RR 0.81, 95% CI 0.74 to 0.90, *p* < 0.001) [11].

Biological sex is increasingly recognized as an essential factor driving susceptibility, pathophysiology, outcomes, and response to therapy in clinical studies in neonatology [18,19,20,21]. Male preterm neonates have a higher risk of mortality before hospital discharge, respiratory distress syndrome, BPD, NEC, late-onset sepsis, severe intraventricular hemorrhage, severe ROP, and neurodevelopmental impairment [18,19,20]. In addition, sex-specific effects of interventions in preterm infants are common [18,21,22,23,24,25,26,27]. This has led to a call for the consideration of sex as a biological variable in data collection, analysis, and reporting of studies, as well as for the evaluation of the interaction between treatment and sex by appropriate statistical methods [18]. When the NeOProM investigators analyzed their data disaggregated by sex, they found some differences in relevant outcomes such as NEC or ROP [11]. However, these differences were not “statistically significant” (*p* > 0.05) according to the frequentist null hypothesis significance testing (NHST).

The dominance of NHST *p*-values when comparing two groups in biomedical research is overwhelming [28]. However, a limitation of the NHST is that the failure to reject the null hypothesis (H_0_, no effect) when the *p*-value is below a predefined threshold (typically 0.05) does not mean that we have found evidence supporting H_0_. Conversely, if we reject H_0_ when the *p*-value is above 0.05, this does not necessarily mean that we have found evidence to support the alternative hypothesis (H_1_, there is an effect) [29,30,31,32]. Bayesian statistics is increasingly recognized and applied in biomedical research as an alternative to overcome these and other limitations of NHST [33,34,35,36,37,38,39]. The Bayes factor (BF) is a tool used in Bayesian inference for hypothesis testing as a way to quantify the relative degree of support for a hypothesis in a data set [32,40,41,42,43]. BFs quantify evidence on a continuous scale, allowing for more nuanced conclusions rather than all-or-none (significant vs. non-significant) conclusions, and can help distinguish between evidence of absence (H_0_ should be accepted) and absence of evidence (inconclusive evidence for both H_0_ and H_1_) [32,33,40,41,42,43,44].

Our present objective was to reanalyze the overall and sex-disaggregated results of the NeOProM studies using a Bayesian approach [40,41,45]. The Bayesian framework may provide a wider, and arguably more informative, set of interpretations than that typically provided by a frequentist analysis [33,44].

## 2. Materials and Methods

This study was exempt from obtaining formal institutional review board approval and from the requirement to obtain informed patient consent because it is secondary research of a publicly available data set [11].

The sex-specific data from each of the five studies included in the NeOProM were re-entered into a new database and the values of log risk ratio (logRR) and the corresponding standard error and 95% confidence interval (CI) of each individual study were calculated using COMPREHENSIVE META-ANALYSIS V4.0 software (Biostat Inc., Englewood, NJ, USA). The results were further pooled and analyzed by a Bayesian-model-averaged (BMA) meta-regression [45], a moderation analysis extension to BMA meta-analysis [40,41]. We performed the BMA in R using the RoBMA R package [46]. BMA employs Bayes factors (BFs) and Bayesian model averaging to evaluate the likelihood of the data under the combination of models assuming the presence vs. the absence of the meta-analytic effect, heterogeneity, and moderation [40,41,45]. The BF_10_ is the ratio of the probability of the data under H_1_ over the probability of the data under H_0_. We used the categories proposed by Lee & Wagenmakers for the interpretation of the BFs [47]. The evidence in favor of H_1_ (BF_10_ > 1) was categorized as weak/inconclusive (1 < BF_10_ < 3), moderate (3 < BF_10_ < 10), strong (10 < BF_10_ < 30), very strong (30 < BF_10_< 100), and extreme (BF_10_ > 100). The evidence in favor of H0 (BF_10_ < 1) was categorized as weak/inconclusive (1/3 < BF_10_ < 1), moderate (1/10 < BF_10_ < 1/3), strong (1/30 < BF_10_ < 1/10), very strong (1/100 < BF_10_ < 1/30), and extreme (BF_10_ < 1/100). The BF_rf_ is the ratio of the probability of the data under the random effects model over the probability of the data under the fixed effect model and BF_mod_ is the ratio of the probability of the data under the moderated models (i.e., by sex differences) vs. the non-moderated models. Furthermore, BFs for the presence vs. absence of the effect at the different level of the moderator (e.g., BF_female_, BF_male_) were calculated using the Savage–Dickey density ratio [48,49]. The categories of strength of the evidence in favor of the random effects (BF_rf_ > 1) or the fixed effect (BF_rf_ < 1), differences by sex (BF_mod_ > 1) or absence of differences by sex (BF_mod_ < 1), and the presence of the effect by sex subgroups (BF_female_ > 1, BF_male_ > 1) or absence of the effect by sex subgroups (BF_female_ < 1, BF_male_ < 1) were similar to those described above for BF_10_.

We used neonatology-specific prior distributions based on the Cochrane Database of Systematic Reviews for logRR, logRR~Student-*t* (µ = 0, σ = 0.18, ν = 3), and tau = inverse-gamma (k = 1.89, θ = 0.30) [50], and tested for the moderation by sex by specifying a Δμi~Normal (µ = 0, σ = 0.18 x s) prior distribution on the difference between each category and the grand mean, with s = 1/2 or s = 1/4 (i.e., the expected difference in each moderator level corresponding to 1/2 or 1/4 of the mean effect size).

## 3. Results

The NeOProM reported 30 categorical outcomes disaggregated by sex. In addition, we pooled the data of positive pressure (with and without endotracheal tube) and supplemental oxygen at 36 weeks’ postmenstrual age (PMA) to obtain an estimate of moderate-to-severe BPD as defined by Jobe & Bancalari [51]. The overall and sex-disaggregated BMA results are shown in Table 1, Table 2 and Table 3. These tables show the analyses with s = 1/4 (i.e., the expected difference in each moderator level corresponding to 1/4 of the mean effect size). Supplementary Tables S1–S3 show the results with s = 1/2 (i.e., the expected difference in each moderator level corresponding to 1/2 of the mean effect size). Supplementary Tables S4–S6 show the original results of the frequentist analysis [11] compared with the results of the Bayesian analysis.

**Figure 1:**
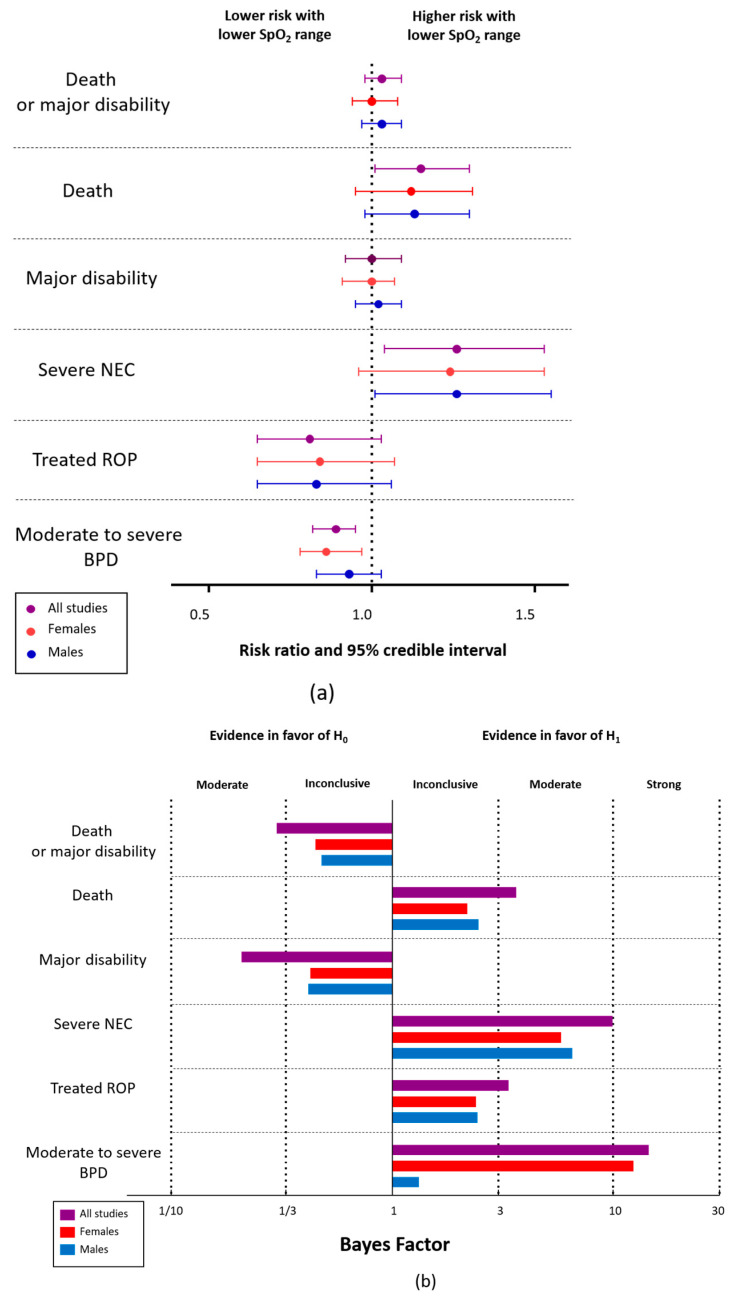
Bayesian reanalysis of the results of the NeOProM study. (**a**) Summary of the overall and sex-disaggregated results. RR > 1 indicates higher risk with lower SpO2 range (85–89% vs. 91–95%); (**b**) Summary of Bayes factors (BFs) calculated through Bayesian-model-averaged (BMA) meta-regression. The BF10 is shown for the overall results and the BFFemale and BFMale for the results disaggregated by sex; BPD: bronchopulmonary dysplasia; NEC: necrotizing enterocolitis; ROP: retinopathy of prematurity.

Regarding the overall results, the Bayesian analysis showed moderate evidence in favor of H_0_ (BF_10_ = 0.30) for the main outcome (death or major disability at 18–24 months’ age corrected for prematurity) (Table 1, Figure 1). When the two components of this major outcome were analyzed separately, the Bayesian analysis showed moderate evidence in favor of H_1_ (BF_10_ = 3.60) for mortality (lower in the group exposed to the high SpO_2_ range) and moderate evidence in favor of H_0_ (BF_10_ = 0.21) for major disability (Table 1, Figure 1). The evidence in favor of H_1_ was also moderate when mortality was defined before 36 weeks’ PMA (BF_10_ = 3.33), and before hospital discharge (BF_10_ = 3.15). The evidence in favor of H_0_ was moderate to inconclusive for the other definitions of major disability used by the investigators (Table 1).

Of the secondary outcomes related to disability, the Bayesian analysis showed moderate evidence in favor of H_0_ for “Bayley-III language and/or cognitive scale < 85” (BF_10_ = 0.22) and “Bayley-III language scale < 85” (BF_10_ = 0.30) (Table 2). With regard to other secondary outcomes, the Bayesian analysis showed that the evidence in favor of H_1_ was very strong for supplemental oxygen at 36 weeks’ PMA (BF_10_ = 99.49, lower rate in lower SpO_2_ group), strong for moderate-to-severe BPD (BF_10_ = 14.44, lower rate in lower SpO_2_ group), strong for severe NEC (BF_10_ = 9.94, lower rate in higher SpO_2_ group), and moderate for treated ROP (BF10 = 3.36, lower rate in lower SpO_2_ group) (Table 3, Figure 1). In addition, the analysis showed moderate evidence in favor of H_0_ for PDA that was medically or surgically treated (BF_10_ = 0.17), oxygen at discharge (BF_10_ = 0.30), and readmission to hospital (BF_10_ = 0.17) (Table 3).

With regard to the results disaggregated by sex, the BMA analysis showed marked differences between the BFs for males and females for two outcomes: supplemental oxygen at 36 weeks’ PMA and moderate-to-severe BPD (Table 3, Figure 1). BMA regression showed that the only outcome with moderate evidence for sex differences (BFmod = 3.41) was moderate-to-severe BPD (Table 3, Figure 1).

To evaluate the robustness of the results, an additional analysis was performed with the s-value set to 1/2. As is shown in Supplementary Tables S1–S3, the use of an s-value of 1/2 did not produce substantial changes in the results.

## 4. Discussion

The NeOProM collaboration have provided the highest quality evidence on what SpO_2_ ranges are most appropriate for extremely preterm infants during the first weeks of life. The main contribution of this Bayesian reanalysis is that it allows an assessment of the strength of this evidence in a way that goes beyond the dichotomous categorization (significant vs. non-significant) of classical frequentist statistics. The Bayesian reanalysis showed that there is moderate evidence in favor of H_0_ (BF_10_ = 0.30) for the primary outcome of the NeOProM trials (death or major disability). In other words, there is moderate evidence of an absence of difference between the two SpO_2_ ranges. This evidence of no difference between the two saturation ranges was confirmed when major disability was analyzed separately from mortality (BF_10_ = 0.21). Interestingly, when mortality was examined separately from major disability, the Bayesian analysis showed moderate evidence (BF_10_ = 3.60) in favor of lower mortality in the group exposed to the higher SpO_2_ range. This confirms the results reported in the frequentist analysis as being “statistically significant” (*p* = 0.01) [11]. Regarding other outcomes, the Bayesian analysis confirmed that exposure to the higher saturation range was associated with a decreased risk of NEC but increased risk of ROP. In addition, the Bayesian analysis showed that the higher SpO_2_ range was associated with a higher risk of moderate-to-severe BPD. Finally, when the results were disaggregated by sex, the BMA regression showed moderate evidence of sex differences in the effects of SpO_2_ ranges on BPD.

In spite of the careful design of the NeOProM trials, there are physiological, technical, and implementation issues with the methods and interventions used in the RCTs that raise questions about the external validity and practical applicability of the findings [52,53,54,55,56,57]. Despite nearly identical protocols with similar pulse oximetry masking for the groups, significant differences in the target were achieved. In both the SUPPORT and COT trials, the median distribution of SpO_2_ was higher than the target. The three BOOST II trials were the most successful in achieving the target for both groups in terms of median values [57]. In addition, the interpretation of the results was complicated by a revision of the calibration software for the study oximeters [56,57]. Furthermore, the two comparison groups may have been more similar than different because of inherent variability in pulse oximeter accuracy, lack of specification of probe placement, and differences in oxygen dissociation curves for fetal and adult hemoglobin [52,53,54,55,56,57]. Therefore, it has been argued that the NeOProM studies may not have been able to separate two true areas of oxygen exposure [52,53,54,55,56,57].

The results of the NeOProM trials were not significant for the primary outcome of death or major disability (RR 1.04, 95% CI 0.98 to 1.09, *p*  =  0.21) [11]. In the case of non-significant or null results, clinicians need to be able to gauge the evidence of the absence of an effect [44]. However, the frequentist approach does not allow us to distinguish whether the null results indicate evidence for the absence of differences between the two saturation ranges or whether they are inconclusive (i.e., absence of evidence). Here, we have shown how this goal of distinguishing between the two situations can be achieved by using BFs. The BF_10_ for the outcome of death or major disability in the NeOProM trials was 0.30. Consequently, the BF_01_ was 3.33 (1/0.3 = 3.33). This means that the data are 3.33 times more likely under H_0_ than under H_1_, which is considered moderate evidence in favor of H0 (no differences between the two saturation ranges).

Despite the limitations mentioned above, the RCTs included in the NeOProM collaboration showed differences between the two saturation ranges in several key outcomes, including mortality. The SUPPORT trial, the first of the NeOProM collaboration to report in-hospital outcomes, reported no difference in the composite primary outcome of death or ROP, but showed evidence that targeting SpO_2_ in the lower range (85% to 89%) was associated with an unanticipated higher mortality rate (RR 1.27; 95% CI, 1.01 to 1.60; *p* = 0.04) [13]. A subsequent safety meta-analysis of the SUPPORT trial along with the three BOOST II trials reported significantly lower mortality in the higher-target group (91% to 95%). As a result, enrollment in two of the BOOST II trials was stopped early because further enrollment could cause harm to participants [58]. Finally, the NeOProM confirmed the higher mortality associated with the lower SpO_2_ range (RR 1.17, 95% CI 1.04 to 1.31, *p*  =  0.01) [11]. The present Bayesian reanalysis showed that the evidence for this finding was moderate (BF_10_ = 3.60). In addition, the Bayesian analysis showed that the evidence was moderate to strong for increased rates of severe NEC but lower rates of severe ROP and moderate-to-severe BPD in the group exposed to the lower SpO_2_ range. Differences in BPD are difficult to interpret because the definition of BPD is based on the need for oxygen and/or respiratory support [51]. It is plausible that if the target saturation is higher, there is a greater likelihood that oxygen will be required to reach that target. Interestingly, the Bayesian analysis showed moderate evidence of no difference between the two saturation ranges when the outcome was mechanical ventilation. This suggests that the development of the more severe forms of lung damage would not be affected by the target SpO_2_ range.

Regarding other complications, both NEC and ROP are two conditions that neonatologists strive to prevent because they have a major impact on the outcome of prematurity. The fact that one is associated with the low SpO_2_ range and the other with the high SpO_2_ range raises the clinical dilemma of accepting higher ROP rates to reduce both mortality and NEC rates. A growing number of observational studies have reported an increase in the rate of severe ROP in association with the introduction of higher SpO_2_ ranges [59,60,61,62]. However, other investigators have not confirmed this increase in ROP [63,64]. Interestingly, neither the differences in ROP nor the differences in NEC ultimately had an effect on the neurodevelopment of the infants in the NeOProM studies. As mentioned above, Bayesian analysis showed moderate evidence in favor of H_0_ for the major disability outcome. In addition, despite the higher rate of ROP in the group exposed to the high SpO_2_ range, the Bayesian analysis showed inconclusive evidence in favor of H_0_ (BF_10_ = 0.83) for the outcome of visual impairment at 18 to 24 months of age.

The underrepresentation of female participants in adult RCTs is a growing concern because low inclusion rates of women may create a lack of crucial knowledge of the adverse effects and the benefit/risk profile of any given treatment [65]. In the case of RCTs conducted in the neonatal population, it appears very unlikely that an imbalance in the inclusion of one of the sexes may occur, but it should be noted that the baseline risk of morbidity and mortality is different for males and females [18,19,20]. Therefore, reporting sex-stratified outcomes for both efficacy and adverse events is of high importance [18,21]. When we analyzed the potential sex differences in the various outcomes of the NeOProM studies, we found that there were marked differences between males and females in the strength of evidence for moderate-to-severe BPD, and oxygen requirement at 36 weeks’ PMA (Table 3). However, just as it would be wrong to conclude that the presence of a statistically significant (*p* < 0.05) association for males combined with no significance (*p* > 0.05) for females implies that there is a sex difference [66], we cannot conclude that the presence of evidence supporting H_1_ for one sex and H_0_ for the other is evidence in favor of a difference [67]. When we tested, using BMA-regression, the possible interaction of biological sex with the different outcomes, we found that the evidence in favor of H_1_ was moderate for the outcome of moderate-to-severe BPD, but inconclusive or in favor of H_0_ (absence of sex differences) for the rest of the outcomes.

The NeOProM project is a major achievement and a milestone in international neonatal research collaboration and has had a profound impact on clinical practice. The potential association of the lower SpO_2_ range (85–89%) with increased mortality and development of NEC led many NICUs worldwide to implement saturation ranges close to the high ranges studied in the trials (91–95%), as recommended by scientific panels and organizations [68,69,70]. However, it should be noted that preterm infants probably have individually different susceptibility to the damage caused by either hypoxia or hyperoxia [52,53,54,55,56,57]. Factors such as perinatal and neonatal comorbidity, gestational and postnatal age, growth, or therapeutic interventions may have an impact on the severity and extent of hypoxic or oxidative stress. Therefore, is unlikely that a single narrow SpO_2_ range can be found that would be safe for all extremely preterm infants [52,53,54,55,56,57]. Nevertheless, it does not appear that the efforts of the neonatal research community will be directed towards conducting new RCTs on SpO_2_ limits. In a search of https://clinicaltrials.gov/ (accessed on 10 January 2024), we could not find any ongoing RCT focusing on SpO_2_ limits in extremely preterm infants outside of the birth resuscitation period.

In conclusion, the present Bayesian reanalysis of the NeOProM trials confirmed that there is moderate evidence that exposure to a SpO_2_ range of 85–89% versus a range of 91–95% is associated with a higher mortality rate in extremely preterm infants. There is strong evidence that the higher SpO_2_ range is associated with a lower rate of severe NEC and moderate evidence that it is associated with a higher rate of severe ROP. Finally, Bayesian reanalysis showed strong evidence for an association between a higher SpO_2_ range and BPD. This association was more apparent in females than in males, suggesting the presence of sex differences in pulmonary susceptibility to oxygen supplementation in extremely preterm infants. The Bayesian approach may provide a new perspective on the scientific evidence from RCTs and meta-analyses, and can help in assessing the strength of evidence that supports clinical decisions.

## Figures and Tables

**Table 1:** Bayesian-model-averaged (BMA) regression of the outcome death and/or major disability in the NeOProM trials.

Outcome	All			Female			Male			BF_10_	BF_rf_	BF_mod_	BF_Female_	BF_Male_
	RR	95% CrI		RR	95% CrI		RR	95% CrI						
		L	U		L	U		L	U					
Death or major disability (primary analysis)	1.03	0.98	1.09	1.00	0.94	1.08	1.03	0.97	1.09	0.30	0.09	0.76	0.45	0.48
Death or major disability (supportive analysis)	1.04	0.98	1.10	1.00	0.93	1.09	1.03	0.98	1.10	0.35	0.12	0.88	0.52	0.59
Death or major disability (secondary analysis)	1.05	0.97	1.14	1.02	0.93	1.14	1.04	0.96	1.14	0.51	0.16	0.75	0.53	0.55
Death or major disability (trialist defined)	1.07	0.99	1.14	1.02	0.93	1.14	1.06	0.99	1.15	1.04	0.18	1.06	0.85	1.19
Major disability (primary analysis)	1.00	0.92	1.09	0.98	0.91	1.07	1.02	0.95	1.09	0.21	0.17	0.84	0.43	0.42
Major disability (supportive analysis)	1.01	0.93	1.10	0.98	0.91	1.07	1.02	0.95	1.10	0.21	0.17	0.92	0.46	0.46
Major disability (secondary analysis)	0.97	0.85	1.11	0.98	0.86	1.10	1.00	0.88	1.11	0.38	0.35	0.89	0.54	0.53
Major disability (trialist defined)	1.03	0.94	1.14	0.99	0.90	1.11	1.04	0.96	1.14	0.32	0.22	1.14	0.57	0.63
Death prior to 18–24 months’ age corrected for prematurity	1.15	1.01	1.30	1.12	0.95	1.31	1.13	0.98	1.30	3.60	0.50	0.84	2.17	2.45
Death prior to 36 weeks’ postmenstrual age	1.15	1.01	1.32	1.13	0.95	1.32	1.14	0.97	1.33	3.33	0.50	0.86	2.08	2.26
Death prior to discharge	1.14	1.01	1.29	1.12	0.94	1.29	1.13	0.98	1.30	3.15	0.45	0.88	1.96	2.22

BF: Bayes factor; CrI: credible interval; L: lower limit; RR: risk ratio; U: upper limit. RR > 1 indicates higher risk with lower SpO_2_ range (85–89% vs. 91–95%).

**Table 2:** Bayesian-model-averaged (BMA) regression of the outcomes related to neurodevelopmental impairment in the NeOProM trials.

Outcome	All			Female			Male			BF_10_	BF_rf_	BF_mod_	BF_Female_	BF_Male_
	RR	95% CrI		RR	95% CrI		RR	95% CrI						
		L	U		L	U		L	U					
Cerebral palsy with GMFCS ≥ 2	1.01	0.81	1.26	1.01	0.84	1.23	1.01	0.84	1.22	0.55	0.59	0.95	0.67	0.66
Severe visual impairment (trialist defined)	1.05	0.75	1.52	1.02	0.77	1.44	1.04	0.78	1.45	0.83	1.00	1.02	0.89	0.90
Deafness requiring hearing aids or worse	1.01	0.77	1.34	1.00	0.79	1.29	1.02	0.81	1.30	0.67	0.97	1.03	0.77	0.76
Bayley-III language and/or cognitive scale < 85	1.00	0.92	0.92	0.99	0.91	1.07	1.01	0.94	1.09	0.22	0.16	0.77	0.40	0.39
Bayley-III cognitive scale < 85	1.04	0.91	1.20	1.02	0.91	1.18	1.02	0.92	1.17	0.41	0.43	0.86	0.54	0.53
Bayley-III language scale < 85	1.03	0.94	1.13	0.99	0.91	1.11	1.03	0.95	1.12	0.30	0.30	0.93	0.54	0.51
Bayley-III language or cognitive scale < 70	0.96	0.81	1.12	0.98	0.82	1.11	0.98	0.83	1.11	0.48	0.51	0.91	0.58	0.58
Bayley-III cognitive scale < 70	1.02	0.82	1.30	1.02	0.84	1.27	1.01	0.84	1.25	0.58	1.00	0.97	0.68	0.67
Bayley-III language scale < 70	1.01	0.85	1.20	1.00	0.86	1.16	1.01	0.88	1.17	0.43	0.43	0.92	0.56	0.57

BF: Bayes factor; GMFCS: Gross Motor Function Classification System; CrI: credible interval; L: lower limit; RR: risk ratio; U: upper limit. RR > 1 indicates higher risk with lower SpO2 range (85–89% vs. 91–95%).

**Table 3:** Bayesian-model-averaged (BMA) regression of secondary outcomes in the NeOProM trials.

Outcome	All			Female			Male			BF_10_	BF_rf_	BF_mod_	BF_Female_	BF_Male_
	RR	95% CrI		RR	95% CrI		RR	95% CrI						
		L	U		L	U		L	U					
PDA medically or surgically treated	1.01	0.96	1.07	1.00	0.94	1.06	1.01	0.96	1.07	0.17	0.05	0.60	0.30	0.30
PDA surgically treated	1.13	0.97	1.32	1.10	0.94	1.33	1.09	0.93	1.32	1.35	0.38	0.90	1.06	1.06
Severe NEC	1.26	1.04	1.53	1.24	0.96	1.53	1.26	1.01	1.55	9.94	0.43	0.99	5.82	6.52
Treated ROP	0.81	0.65	1.03	0.84	0.65	1.07	0.83	0.65	1.06	3.36	8.97	0.98	2.40	2.43
Positive airway press with ETT at 36 weeks’ PMA	0.99	0.82	1.19	0.97	0.83	1.15	1.02	0.86	1.18	0.47	0.71	1.15	0.73	0.72
Positive airway press w/o ETT at 36 weeks’ PMA	1.10	0.81	1.02	0.92	0.80	1.05	0.94	0.82	1.06	1.32	0.70	0.83	1.07	0.99
Suppl. O_2_ w/o positive press. at 36 weeks’ PMA	0.83	0.75	0.92	0.82	0.73	0.93	0.84	0.75	0.95	99.49	0.21	0.93	49.31	31.11
Moderate-to-severe BPD	0.89	0.82	0.95	0.85	0.78	0.97	0.93	0.83	1.03	14.44	1.10	3.41	12.32	1.32
Discharged home on oxygen	1.01	0.90	1.13	1.00	0.91	1.11	1.01	0.92	1.12	0.30	0.38	0.83	0.46	0.46
Readmission to hospital	1.01	0.95	1.08	0.98	0.92	1.05	1.02	0.96	1.09	0.17	0.25	0.91	0.43	0.43

BF: Bayes factor; BPD: bronchopulmonary dysplasia; CrI: credible interval; ETT: endotracheal tube; L: lower limit; NEC: necrotizing enterocolitis; PDA: patent ductus arteriosus; PMA: postmenstrual age; press: pressure; ROP: retinopathy of prematurity; RR: risk ratio; Suppl.: supplementary; U: upper limit. RR > 1 indicates higher risk with lower SpO2 range (85–89% vs. 91–95%).

## Data Availability

All data relevant to the study are included in the article or uploaded as Supplementary Information. Additional data are available upon reasonable request.

## Supplementary Materials

The following supporting information can be downloaded at: https://www.mdpi.com/article/10.3390/antiox13050509/s1, Table S1: Bayesian-model-averaged (BMA) regression of the outcome death and/or major disability in the NeOProM trials (analyses with s = 1/2); Table S2: Bayesian-model-averaged (BMA) regression of the outcomes related to neurodevelopmental impairment in the NeOProM trials (analyses with s = 1/2); Table S3: Bayesian-model-averaged (BMA) regression of secondary outcomes in the NeOProM trials (analyses with s = 1/2). Table S4: Comparison between the Bayes Factor (BF) values of the Bayesian-model-averaged (BMA) analysis and the *p*-values of the original NeOProM frequentist analysis for the outcomes of death and/or major disability; Table S5: Comparison between the Bayes Factor (BF) values of the Bayesian-model-averaged (BMA) analysis and the *p*-values of the original NeOProM frequentist analysis for the outcomes related to neurodevelopmental impairment; Table S6: Comparison between the Bayes Factor (BF) values of the Bayesian-model-averaged (BMA) analysis and the *p*-values of the original NeOProM frequentist analysis for the secondary outcomes.
