# Supplementary material for: A Bayesian Reanalysis of the Overall and Sex-Disaggregated Results of the Neonatal Oxygenation Prospective Meta-Analysis (NeOProM)
Source: Antioxidants (Basel). 2024 Apr 24;13(5):509. doi: 10.3390/antiox13050509 (PMC11117690; doi:10.3390/antiox13050509)
Supplement: Supplementary file 1 [file antioxidants-13-00509-s001.zip › antioxidants-2937255-supplementary.pdf]

Supplementary Materials:

A Bayesian reanalysis of the overall and sex-disaggregated results of the NeOProM studies

Maurice Jacob Huizing, Tamara Maria Hundscheid, František Bartoš, Eduardo Villamor.

Table S1. Bayesian model averaged (BMA) regression of the outcome death and/or major disability in the NeOProM trials (analyses with $s = 1/2$)

Table S2. Bayesian model averaged (BMA) regression of the outcomes related to neurodevelopmental impairment in the NeOProM trials (analyses with $s = 1/2$)

Table S3. Bayesian model averaged (BMA) regression of secondary outcomes in the NeOProM trials (analyses with $s = 1/2$)

Table S4. Comparison between the Bayes Factor (BF) values of the Bayesian model averaged (BMA) analysis and the p-values of the original NeOProM frequentist analysis for the outcomes death and/or major disability.

Table S5. Comparison between the Bayes Factor (BF) values of the Bayesian model averaged (BMA) analysis and the p-values of the original NeOProM frequentist analysis for the outcomes related to neurodevelopmental impairment.

Table S6. Comparison between the Bayes Factor (BF) values of the Bayesian model averaged (BMA) analysis and the p-values of the original NeOProM frequentist analysis for the secondary outcomes.

Table S1. Bayesian model averaged (BMA) regression of the outcome death and/or major disability in the NeOProM trials (analyses with s = 1/2)

Outcome	All			Female			Male			BF_{10}	BF_{rf}	BF_{mod}	BF_{Female}	BF_{Male}
	RR	95% CrI		RR	95% CrI		RR	95% CrI						
		Lower	Upper		Lower	Upper		Lower	Upper					
Death or major disability (primary analysis)	1.03	0.98	1.09	1.00	0.93	1.09	1.03	0.98	1.10	0.30	0.10	0.47	0.35	0.38
Death or major disability (supportive analysis)	1.04	0.98	1.10	1.00	0.92	1.09	1.04	0.98	1.11	0.35	0.12	0.58	0.42	0.46
Death or major disability (secondary analysis)	1.05	0.97	1.14	1.02	0.92	1.14	1.04	0.96	1.14	0.51	0.16	0.49	0.45	0.48
Death or major disability (trialist defined)	1.07	0.99	1.14	1.02	0.91	1.13	1.07	0.99	1.15	1.00	0.18	0.78	0.71	1.17
Major disability (primary analysis)	1.00	0.92	1.09	0.98	0.89	1.07	1.02	0.94	1.11	0.21	0.17	0.58	0.34	0.34
Major disability (supportive analysis)	1.01	0.93	1.09	0.97	0.89	1.07	1.03	0.95	1.12	0.21	0.17	0.67	0.40	0.39
Major disability (secondary analysis)	0.97	0.85	1.11	0.97	0.82	1.12	1.00	0.87	1.14	0.39	0.36	0.69	0.48	0.47
Major disability (trialist defined)	1.03	0.93	1.14	0.97	0.87	1.11	1.05	0.96	1.16	0.31	0.22	1.00	0.57	0.61
Death prior to 18-24 months' age corrected for prematurity	1.15	1.01	1.30	1.12	0.93	1.32	1.14	0.98	1.32	3.50	0.51	0.62	1.75	1.99
Death prior to 36 weeks' postmenstrual age	1.15	1.01	1.32	1.13	0.92	1.34	1.14	0.97	1.34	3.26	0.51	0.65	1.75	2.07
Death prior to discharge	1.14	1.01	1.29	1.11	0.91	1.30	1.14	0.98	1.32	3.04	0.46	0.67	1.50	1.94

The table show the analyses with s = 1/2 (i.e., the expected difference of each moderator level corresponding to 1/2 of the mean effect size).
BF: Bayes factor; CrI: credible interval; RR: risk ratio.
RR>1 indicates higher risk with lower SpO_2 range (85-89% vs. 91-95%).

Table S2. Bayesian model averaged (BMA) regression of the outcomes related to neurodevelopmental impairment in the NeOProM trials (analyses with $s = 1/2$)

Outcome	All			Female			Male			BF_{10}	BF_{rf}	BF_{mod}	BF_{Female}	BF_{Male}
	RR	95% CrI		RR	95% CrI		RR	95% CrI						
		Lower	Upper		Lower	Upper		Lower	Upper					
Cerebral palsy with GMFCS ≥ 2	1.01	0.81	1.26	1.01	0.82	1.26	1.00	0.82	1.23	0.55	0.59	0.84	0.63	0.64
Severe visual impairment (trialist defined)	1.05	0.74	1.51	1.00	0.73	1.45	1.06	0.77	1.51	0.83	0.99	1.08	0.95	0.94
Deafness requiring hearing aids or worse	1.00	0.76	1.32	0.97	0.74	1.27	1.04	0.80	1.32	0.67	0.96	1.09	0.81	0.79
Bayley-III language and/or cognitive <85	1.00	0.92	1.09	0.98	0.90	1.08	1.02	0.93	1.11	0.22	0.16	0.52	0.33	0.32
Bayley-III cognitive <85	1.04	0.91	1.20	1.01	0.88	1.19	1.03	0.90	1.19	0.41	0.44	0.65	0.48	0.48
Bayley-III language <85	1.03	0.94	1.13	0.99	0.88	1.11	1.04	0.95	1.15	0.29	0.31	0.70	0.45	0.47
Bayley-III language or cognitive <70	0.96	0.81	1.13	0.97	0.80	1.14	0.98	0.83	1.15	0.49	0.52	0.73	0.55	0.52
Bayley-III cognitive <70	1.02	0.82	1.30	1.03	0.83	1.30	1.00	0.82	1.25	0.58	1.00	0.89	0.64	0.64
Bayley-III language <70	1.00	0.84	1.19	0.99	0.83	1.17	1.01	0.86	1.19	0.43	0.44	0.76	0.55	0.53

The table show the analyses with s = 1/2 (i.e., the expected difference of each moderator level corresponding to 1/2 of the mean effect size).
BF: Bayes factor; CrI: credible interval; GMFCS: Gross Motor Function Classification System; RR: risk ratio.
RR>1 indicates higher risk with lower SpO_2 range (85-89% vs. 91-95%).

Table S3. Bayesian model averaged (BMA) regression of secondary outcomes in the NeOProM trials (analyses with s = 1/2)

Outcome	All			Female			Male			BF_{10}	BF_{rf}	BF_{mod}	BF_{Female}	BF_{Male}
	RR	95% CrI		RR	95% CrI		RR	95% CrI						
		Lower	Upper		Lower	Upper		Lower	Upper					
PDA medically or surgically treated	1.01	0.95	1.08	1.00	0.94	1.07	1.01	0.95	1.08	0.17	0.05	0.35	0.23	0.23
PDA surgically treated	1.13	0.97	1.33	1.11	0.93	1.35	1.09	0.90	1.33	1.35	0.39	0.71	1.03	0.92
Severe NEC	1.26	1.04	1.53	1.22	0.92	1.53	1.27	1.02	1.59	9.31	0.43	0.92	3.65	6.53
Treated ROP	0.81	0.65	1.03	0.85	0.65	1.12	0.82	0.63	1.07	3.33	8.78	0.90	1.90	2.25
Positive airway pressure with ETT at 36 weeks' PMA	0.99	0.82	1.19	0.93	0.77	1.14	1.06	0.87	1.25	0.48	0.65	1.42	0.89	0.84
Positive airway pressure without ETT at 36 weeks' PMA	0.91	0.81	1.02	0.92	0.79	1.06	0.94	0.81	1.09	1.33	0.72	0.60	0.97	0.83
Supplemental oxygen without positive airway pressure at 36 weeks' PMA	0.83	0.75	0.92	0.81	0.71	0.92	0.84	0.75	0.96	99.63	0.21	0.72	40.79	11.31
Moderate to severe BPD	0.89	0.82	0.95	0.84	0.76	0.95	0.95	0.83	1.04	19.74	0.70	4.43	17.44	0.54
Discharged home on oxygen	1.01	0.90	1.13	0.99	0.88	1.12	1.02	0.90	1.14	0.30	0.39	0.59	0.40	0.40
Readmission to hospital	1.01	0.95	1.08	0.98	0.91	1.06	1.03	0.96	1.10	0.17	0.25	0.60	0.32	0.33

The table show the analyses with s = 1/2 (i.e., the expected difference of each moderator level corresponding to 1/2 of the mean effect size).
BF: Bayes factor; BPD: bronchopulmonary dysplasia; CrI: credible interval; ETT: endotracheal tube; NEC: necrotizing enterocolitis; PDA: patent ductus arteriosus; PMA: postmenstrual age; ROP: retinopathy of prematurity; RR: risk ratio.
RR>1 indicates higher risk with lower SpO_2 range (85-89% vs. 91-95%).

Table S4. Comparison between the Bayes Factor (BF) values of the Bayesian model averaged (BMA) analysis and the p-values of the original NeOProM frequentist analysis for the outcomes death and/or major disability.

Outcome	**Overall**		**Female**		**Male**		**Interaction of sex**	
	BF_{10}	*Frequentist p-value*	BF_{Female}	*Frequentist p-value*	BF_{Male}	*Frequentist p-value*	BF_{mod}	*Frequentist p-value*
Death or major disability (primary analysis)	0.30	*0.210*	0.45	*0.724*	0.48	*0.150*	0.76	*0.543*
Death or major disability (supportive analysis)	0.35	*0.200*	0.52	*0.838*	0.59	*0.106*	0.88	*0.476*
Death or major disability (secondary analysis)	0.51	*0.156*[a]	0.53	*0.489*	0.55	*0.217*	0.75	*0.852*
Death or major disability (trialist defined)	1.04	*0.031*[a]	0.85	*0.632*	1.19	*0.030*	1.06	*0.383*
Major disability (primary analysis)	0.21	*0.950*	0.43	*0.479*	0.42	*0.521*	0.84	*0.338*
Major disability (supportive analysis)	0.21	*0.870*	0.46	*0.471*	0.46	*0.421*	0.92	*0.333*
Major disability (secondary analysis)	0.38	*0.661*[a]	0.54	*0.418*	0.53	*0.857*	0.89	*0.529*
Major disability (trialist defined)	0.32	*0.367*[a]	0.57	*0.589*	0.63	*0.186*	1.14	*0.258*
Death prior to 18-24 months' age corrected for prematurity	3.60	*0.010*	2.17	*0.130*	2.45	*0.029*	0.84	*0.840*
Death prior to 36 weeks' postmenstrual age	3.33	*0.010*	2.08	*0.156*	2.26	*0.037*	0.86	*0.814*
Death prior to discharge	3.15	*0.010*	1.96	*0.208*	2.22	*0.024*	0.88	*0.648*

BF: Bayes factor; CrI: credible interval; RR: risk ratio.

[a]P-value not available in the original study and calculated by means of a random effects meta-frequentist meta-analysis.

Table S5. Comparison between the Bayes Factor (BF) values of the Bayesian model averaged (BMA) analysis and the p-values of the original NeOProM frequentist analysis for the outcomes related to neurodevelopmental impairment.

Outcome	**Overall**		**Female**		**Male**		**Interaction of sex**	
	BF_{10}	*Frequentist p-value*	BF_{Female}	*Frequentist p-value*	BF_{Male}	*Frequentist p-value*	BF_{mod}	*Frequentist p-value*
Cerebral palsy with GMFCS ≥ 2	0.55	*0.880*	0.67	*0.826*	0.66	*0.972*	0.95	*0.862*
Severe visual impairment (trialist defined)	0.83	*0.730*	0.89	*0.222*	0.90	*0.161*	1.02	*0.076*
Deafness requiring hearing aids or worse	0.67	*0.790*	0.77	*0.208*	0.76	*0.220*	1.03	*0.094*
Bayley-III language and/or cognitive <85	0.22	*0.920*	0.40	*0.416*	0.39	*0.618*	0.77	*0.449*
Bayley-III cognitive <85	0.41	*0.524*[a]	0.54	*0.540*	0.53	*0.668*	0.86	*0.844*
Bayley-III language <85	0.30	*0.451*[a]	0.54	*0.530*	0.51	*0.353*	0.93	*0.395*
Bayley-III language or cognitive <70	0.48	*0.513*[a]	0.58	*0.548*	0.58	*0.622*	0.91	*0.912*
Bayley-III cognitive <70	0.58	*0.703*[a]	0.68	*0.615*	0.67	*0.729*	0.97	*0.456*
Bayley-III language <70	0.43	*0.947*[a]	0.56	*0.865*	0.57	*0.888*	0.92	*0.901*

BF: Bayes factor; CrI: credible interval; GMFCS: Gross Motor Function Classification System; RR: risk ratio.
[a]P-value not available in the original study and calculated by means of a random effects meta-frequentist meta-analysis.

Table S6. Comparison between the Bayes Factor (BF) values of the Bayesian model averaged (BMA) analysis and the p-values of the original NeOProM frequentist analysis for the secondary outcomes.

Outcome	**Overall**		**Female**		**Male**		**Interaction of sex**	
	BF_{10}	*Frequentist p-value*	BF_{Female}	*Frequentist p-value*	BF_{Male}	*Frequentist p-value*	BF_{mod}	*Frequentist p-value*
PDA medically or surgically treated	0.17	*0.710*	0.30	*0.853*	0.30	*0.521*	0.60	*0.722*
PDA surgically treated	1.35	*0.046*	1.06	*0.141*	1.06	*0.156*	0.90	*0.989*
Severe NEC	9.94	*0.003*	5.82	*0.243*	6.52	*0.004*	0.99	*0.384*
Treated ROP	3.36	*<0.001*	2.40	*0.115*	2.43	*<0.001*	0.98	*0.212*
Positive airway pressure with ETT at 36 weeks' PMA	0.47	*0.966*[a]	0.73	*0.118*	0.72	*0.273*	1.15	*0.055*
Positive airway pressure without ETT at 36 weeks' PMA	1.32	*0.065*[a]	1.07	*0.032*	0.99	*0.282*	0.83	*0.369*
Supplemental oxygen without positive airway pressure at 36 weeks' PMA	99.49	*<0.001*	49.31	*<0.001*	31.11	*0.025*	0.93	*0.213*
Moderate to severe BPD	14.44	*0.001*[a]	12.32	*<0.001*[a]	1.32	*0.101*[a]	3.41	*0.037*[a]
Discharged home on oxygen	0.30	*0.866*[a]	0.46	*0.783*	0.46	*0.638*	0.83	*0.536*
Readmission to hospital	0.17	*0.640*	0.43	*0.514*	0.43	*0.437*	0.91	*0.286*

BF: Bayes factor; BPD: bronchopulmonary dysplasia; CrI: credible interval; ETT: endotracheal tube; NEC: necrotizing enterocolitis; PDA: patent ductus arteriosus; PMA: postmenstrual age; ROP: retinopathy of prematurity; RR: risk ratio.

[a]P-value not available in the original study and calculated by means of a random effects meta-frequentist meta-analysis.
